# Multi-year dietary and parasitological investigations of invasive Egyptian geese (*Alopochen aegyptiaca*) in central Germany

**DOI:** 10.1016/j.ijppaw.2026.101288

**Published:** 2026-09-12

**Authors:** Anna V. Schantz, Robin Stutz, Anne Steinhoff, Norbert Peter, Anna Thomas, Dorian D. Dörge, Sven Klimpel

**Affiliations:** a Institute for Ecology, Evolution and Diversity, Goethe-University, Max-von-Laue-Str. 13, Frankfurt/Main, D-60438, Germany; b Senckenberg Biodiversity and Climate Research Centre, Senckenberg Gesellschaft für Naturforschung, Senckenberganlage 25, Frankfurt/Main, D-60325, Germany

**Keywords:** Egyptian goose, *Alopochen aegyptiaca*, Invasive species, Parasites, Diet

## Abstract

The Egyptian goose *Alopochen aegyptiaca*, native to Africa, was introduced to Europe between the 17th and 20th centuries as an ornamental and park bird. Since then, it has spread widely and has reached very high population densities in several countries, including Germany. In 2017, it was listed as an invasive alien species of Union concern, because its continued spread may have ecological impacts. To investigate its feeding ecology and parasite fauna, 100 Egyptian geese from the Wetterau region in Hesse, Germany, were examined over four years. Their diet consisted mainly of plant material, particularly corn, grasses and grains, but also included animal components such as mussel fragments and annelids. Parasitological analyses identified six ectoparasite and eleven endoparasite species. *Anaticola asymmetricus*, *Anatoecus dentatus*, *Echinostoma revolutum*, *Notocotylus attenuatus* and *Polymorphus minutus* occurred in all study years and were classified as key species. The dietary results indicate opportunistic use of abundant and easily accessible resources, with temporary preferences for certain resources in the study area. The parasite fauna was highly diverse and substantially expands the known parasite spectrum of the Egyptian goose, including seven first records for Europe. Combining dietary and parasitological analyses demonstrated that animal components constitute a certain proportion of the diet and enables inferences about earlier feeding events. Food availability and parasite occurrence are likely influenced by ecosystem conditions, the presence of potential intermediate and definitive hosts and climatic conditions. Overall, this study provides a comprehensive overview of the feeding ecology and parasite fauna of Egyptian geese in the study area and broadens current knowledge of their parasites in Germany and Europe.

## Highlights

• Study of 100 Egyptian geese over a 4-year period in a defined area in Germany.
• Dietary and parasitological analyses were combined.
• Diet consisted mainly of plants but included small amounts of animal components.
• Dissection resulted in identification of 6 ectoparasites and 11 endoparasites.
• This resulted in 7 European first records for the Egyptian goose.

## Graphical abstract

**Figure:**
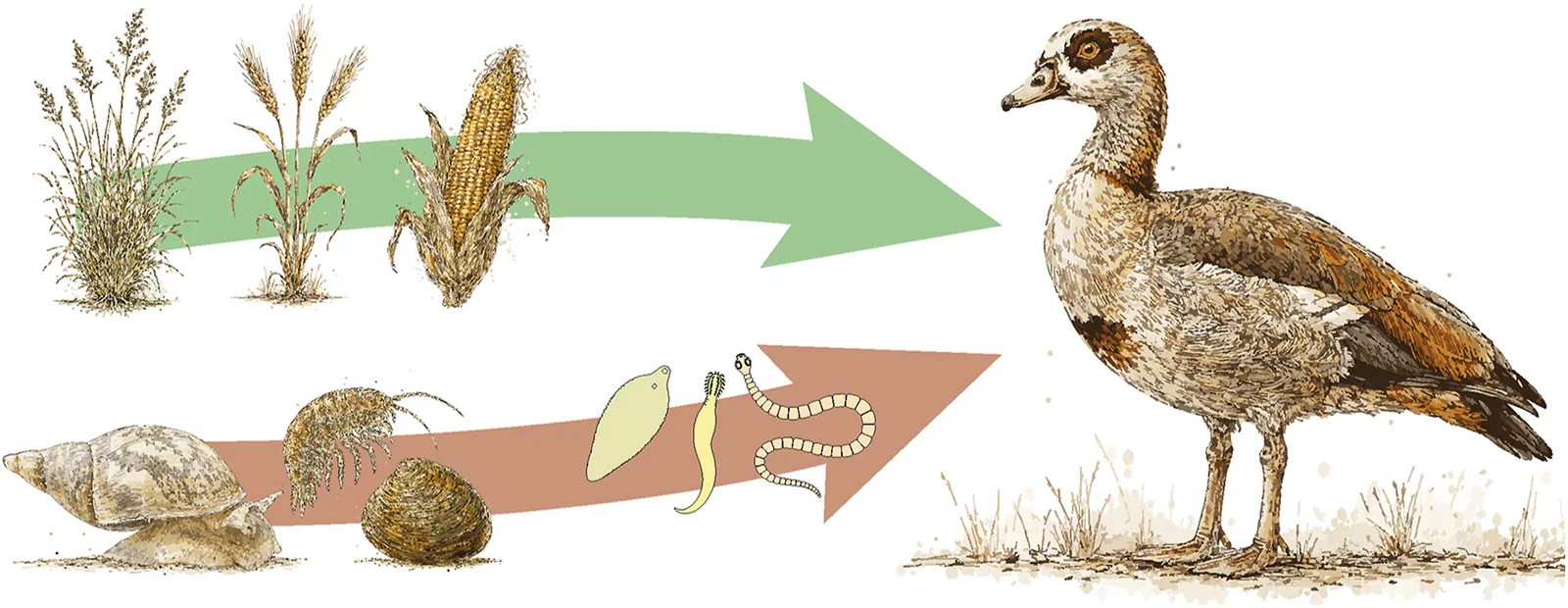

## 1 Introduction

The Egyptian goose *Alopochen aegyptiaca* is taxonomically classified within the family Anatidae and the subfamily Tadorninae (e.g. Scharon, 2015; Abdelsabour-Khalaf and Ahmed, 2020). From its native African range, the Egyptian goose was introduced to various European countries between the 17th and 20th centuries as an ornamental and park bird (Sutherland and Allport, 1991; Gyimesi and Lensink, 2012). In 1967, wild pairs of Egyptian geese bred in the Netherlands for the first time. Since the 1980s, the species has continuously expanded toward Germany and Denmark (Banks et al., 2008; Gyimesi and Lensink, 2012). At the same time, additional populations originating from Brussels developed in Belgium and France. In the following years, the Egyptian goose population within Europe grew exponentially (Gyimesi and Lensink, 2012).

Since August 2, 2017, the Egyptian goose has been listed as an invasive alien species of Union concern (Regulation (EU) No. 1143/2014) and is considered established in Germany. The Egyptian goose is one of the fastest-spreading non-native waterfowl (Banks et al., 2008). In Germany, it is now more widespread than the native greylag goose *Anser anser*. Since 2009, the number of breeding pairs of Egyptian geese has more than doubled (Baudach et al., 2025). High numbers of Egyptian geese are also recorded in Hesse, and the annually increasing hunting bags, particularly in North Rhine-Westphalia, Lower Saxony, and Hesse, indicate a continuing increase in population size (Baudach et al., 2025; Gerlach et al., 2025). A nationwide survey of breeding populations for the period from 2017 to 2022 estimated a total of 11,000–15,500 breeding pairs of Egyptian geese in Germany (Gerlach et al., 2025).

The introduction and spread of invasive alien species such as the Egyptian goose can be associated with various ecological impacts, including competition for breeding sites and food resources, as well as hybridization with other waterfowl species. Hybridization has been documented with both other alien species, such as the Canada goose *Branta canadensis*, and native species. Unpaired Egyptian geese appear to be prone to hybridization with species of the Tadorna group (Weller, 1969; Kolbe, 1990; Curtis et al., 2007; Banks et al., 2008; Gyimesi and Lensink, 2012). In addition, damage to agricultural crops can lead to economic conflicts. Among other places, Egyptian geese forage in grain fields and can thereby reduce crop yields (e.g. Blair et al., 2000; Conover, 2002; Mangnall and Crowe, 2002). Corresponding impacts of large Egyptian goose populations have already been documented in their native African range (Kolbe, 1990; Conover, 2002; Mangnall and Crowe, 2002; Huysentruyt et al., 2020). Conflicts with local communities may also arise, as Egyptian geese are often perceived as aggressive and can contaminate public parks with their excrement (Kolbe, 1990; Bomford, 2003; Banks et al., 2008). In this context, there is also a potential risk of transmission of parasites and other pathogens (e.g. Avery, 1966; Fischer et al., 2023a, 2023b).

To date, only a few studies have provided a comprehensive overview of the parasite fauna and parasite diversity of Egyptian geese in Europe (e.g. Bentele et al., 2026; Fischer et al., 2023b; Prüter et al., 2020). However, data on temporal changes in parasite occurrence and on the species' feeding ecology are lacking. The present study examines changes in the feeding ecology and parasite diversity of invasive Egyptian geese over a four-year period in a defined study area in Hesse. To the authors’ knowledge, this is the first study to examine both aspects together as part of a multi-year monitoring program.

## 2 Material and methods

### 2.1 Sampling

Over a four-year period, 25 Egyptian geese were studied each year in the Wetterau region near the towns of Niederwöllstadt and Dortelweil (Hesse, Germany) as part of a comparative study of their feeding ecology and parasite diversity. Sampling took place exclusively in rural areas on farmland, including corn fields and along the river Nidda, where the animals were grazing all year round. All animals were collected during the fall and winter months and were hunted legally between September 1, 2021, and January 15, 2025 (during the legally designated hunting season for Egyptian geese in Hesse). Until laboratory analysis, the animals were stored in plastic bags at −20°C. In total, 100 Egyptian geese from four seasons (2021/2022, 2022/2023, 2023/2024, 2024/2025) were examined.

### 2.2 External examination

At the beginning of the study, biometric data were collected for each specimen, following the guidelines in Baldwin et al. (1931). The animals were also examined for anomalies and ectoparasitic infestations. Any parasitic individuals found were preserved in 70% EtOH for further morphological identification.

### 2.3 Diet analysis and examination for endoparasites via necropsy

The necropsy of the animals followed the guidelines for bird preparation outlined in Storch and Welsch (2014). Sex determination was performed during dissection based on the presence or absence of the male characteristics testes and Bulla syringealis. To analyze feeding ecology, the crop contents were weighed and sorted by major taxonomic groups. The individual organs (trachea, heart, crop, proventriculus, gizzard, small intestine, large intestine, cecum, cloaca) were examined under a binocular microscope for parasitic stages. Any endoparasites found were stored in 70% EtOH for further morphological identification and in absolute EtOH for genetic identification.

### 2.4 Species identification and statistics

To investigate feeding ecology, the previously weighed crop contents were sorted and assigned to the smallest possible taxon based on morphological characteristics. To quantitatively assess the individual food resources, the frequency of occurrence [F%] and the respective weight percentage [W%] were calculated according to the methods of Klimpel et al. (2019).

Ectoparasites and endoparasites were identified morphologically using identification guides (Yamaguti, 1938; Schultz, 1940; Van Cleave, 1947; von Kéler, 1960; Price, 1971; McDonald, 1974; Eichler and Vasjukova, 1980, 1981; Kanev, 1994; Gaud and Atyeo, 1996; Muniz-Pereira and Amato, 1998; Price et al., 2003; Silveira et al., 2006; Nowak et al., 2011; Grossi et al., 2014; Köhler, 2015; Tamaru et al., 2015; Marinova, 2017; Hou et al., 2020; Jirsa et al., 2021). In addition, the presence of endoparasites was genetically verified, for this purpose, DNA was extracted using the DNeasy Blood & Tissue Kit from Qiagen (Cat. No. 69506), followed by PCR in a 15 μl reaction volume (7.5 μl Taq 2X Master Mix (VWR), 0.35 μl each of forward and reverse primers, 6.8 μl DNA). The thermocycling settings were based on the primers used (Folmer et al., 1994; Morgan and Blair, 1995; Littlewood and Olson, 2001; Holterman et al., 2006; Nugaraitė et al., 2017) (Table 1). Gel electrophoresis was performed for verification, the samples were purified using the NucleoSpin Gel & PCR Clean-up Kit (Macherey-Nagel, Düren, Germany) and subsequently sent to Seqlab GmbH (Göttingen, Germany) for sequencing. The generated sequences were compared to existing reference sequences in the NCBI database based on query length, query coverage, and percent identity. The sequences with the highest match were uploaded to the NCBI database under the accession numbers SAMN62048472 - SAMN62048477 (BioProject PRJNA1504217).

**Table 1:** Final results of genetic analysis of endoparasites after comparison with the NCBI database based on Query length, Query Cover [%] and Percent Identity [%]; the sequences are uploaded to Genbank NCBI under the corresponding accession number.

	Accession number	Species	Query length	Query Cover [%]	Percent Identity [%]	Accession number of reference sequence	Primer name (forward/reverse)	Primer sequence 5'- > -3' (forward/reverse)	Primer source
Digenea	SAMN62048472	*Echinostoma revolutum*	1133	100	99.82	AY222132.1	WormA/WormB	GCGAATGGCTCATTAAATCAG/CTTGTTACGACTTTTACTTCC	Littlewood and Olson (2001); Nugaraitė et al. (2017)
	SAMN62048473	*Notocotylus* sp.	1211	100	99.17	PP274989.1	BD1/BD2	GTCGTAACAAGGTTTCCGTA/TATGCTTAAATTCAGCGGGT	Morgan and Blair (1995); Nugaraitė et al. (2017)
Nematoda	SAMN62048474	*Capillaria anatis*	986	99	93.89	LC052334.1	988F/1912R	CTCAAAGATTAAGCCATGC/TTTACGGTCAGAACTAGGG	Holterman et al. (2006)
	SAMN62048475	*Hystrichis tricolor*	958	99	99.89	MT141133.1			
	SAMN62048476	*Tetrameres fissispina*	959	99	99.58	EF180077.1			
Acantho-cephala	SAMN62048477	*Polymorphus minutus*	640	98	97.92	EF467865.1	LCO1490/HCO2198	GGTCAACAAATCATAAAGATATTGG/TAAACTTCAGGGTGACCAAAAAATCA	Folmer et al. (1994)

Parasite infestation was evaluated according to the guidelines of Klimpel et al. (2019). For this purpose, the prevalence (P%), the mean (mI) and maximum intensity (maxI), as well as the mean abundance (mA) per parasite species were calculated.

## 3 Result

### 3.1 Morphometric data

Of the 100 Egyptian geese examined, 48 were male and 52 were female. The average total weight was 2350.03 g, the average slaughter weight was 1903.01 g, the average body length was 68.43 cm and the average wingspan was 119.67 cm (Table 2).

**Table 2:** Morphometric data of examined Egyptian geese; mean values are given for males (M), females (F) and both (M + F) for the entire sampling period and each individual sampling year.

Male (M)/Female (F)	Sampling period	Total weight [g]	Carcass weight [g]	Total length [cm]	Wingspan [cm]
**M** (n = 48)	**2021 - 2025**	2625.94	2140.03	70.91	125.16
**F** (n = 52)		2100.65	1688.77	66.19	114.72
**M + F** (N = 100)		2350.03	1903.01	68.43	119.67
**M** (n = 13)	**2021/2022**	2813.33	2275.00	71.28	125.48
**F** (n = 12)		2455.17	1968.50	70.27	122.83
**M + F** (n = 25)		2634.25	2121.75	70.77	124.15
**M** (n = 13)	**2022/2023**	2489.15	2059.58	69.78	128.51
**F** (n = 12)		2173.92	1794.19	66.08	123.53
**M + F** (n = 25)		2337.84	1932.19	68.00	126.23
**M** (n = 11)	**2023/2024**	2554.09	2089.27	70.97	119.68
**F** (n = 14)		1905.00	1497.43	64.27	108.55
**M + F** (n = 25)		2190.60	1757.84	67.22	113.45
**M** (n = 11)	**2024/2025**	2655.00	2138.64	71.77	126.45
**F** (n = 14)		1929.64	1550.00	64.71	107.04
**M + F** (n = 25)		2248.80	1809.00	67.82	115.13

During the study period 2021/2022, the birds examined had a total weight of 2634.25 g, a slaughter weight of 2121.75 g, and a body length of 70.77 cm and were therefore heaviest and biggest, the wingspan was longest during the 2022/2023 study period at 126.23 cm (Table 2). Overall, the morphometric values for male Egyptian geese were higher than those for females.

### 3.2 Dietary analysis

**Fig. 1:**
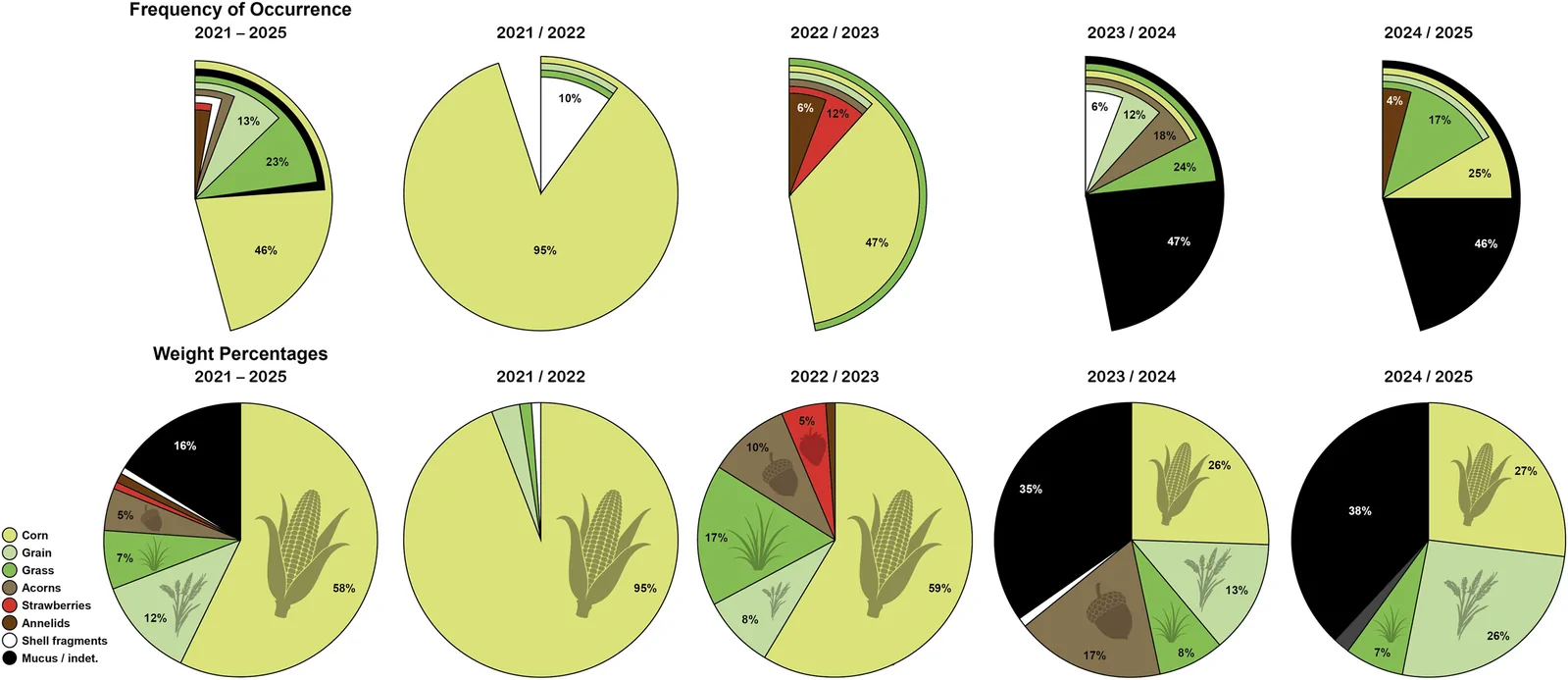
Representation of dietary components including Frequency of occurrence [%] and respective Weight percentage [%] for the entire sampling period and each individual sampling year.

Over the entire study period (2021–2025), 78 out of the 100 Egyptian goose crop samples contained food components. Corn (F = 46.15%), grass (F = 23.08%), and grains (F = 12.82%) were the most common components, along with mucus and unidentified components (F = 24.36%), the weight percentage of corn (W = 57.74%) was the highest (Table 3, Fig. 1). Across the four study periods, corn, grains, and grass were present in all years, nevertheless, the dietary composition varied. Corn was consumed most frequently during the first study period (2021/2022; F = 95.00%), whereas grass was detected more frequently alongside corn in the subsequent study periods. Strawberries were detected only in 2022/2023, with a frequency of occurrence of F = 11.76% and a weight percentage of W = 5.33%, acorns occurred only in the middle two sampling years. Shell fragments and annelids each occurred in only two years of study and even then, with a very low weight percentage of no more than W = 1.87%. In the first sampling year, four different food components were identified, while in subsequent years there were five to six different components.

**Table 3:** Frequency of occurrence F [%] and respective Weight percentage W [%] of major taxonomic groups of crop content for the entire sampling period and each individual sampling year.

	2021-2025		2021/2022		2022/2023		2023/2024		2024/2025	
	N = 78		n = 20		n = 17		n = 17		n = 24	
	**F[%]**	**W[%]**	**F[%]**	**W[%]**	**F[%]**	**W[%]**	**F[%]**	**W[%]**	**F[%]**	**W[%]**
Corn	46.15	57.74	95.00	94.50	47.06	58.79	17.65	25.78	25.00	27.04
Grain	12.82	12.09	10.00	3.27	11.76	8.40	11.76	13.24	16.67	26.27
Grass	23.08	7.01	10.00	1.15	47.06	16.72	23.53	7.81	16.67	6.78
Acorns	6.41	4.96	-	-	11.76	9.83	17.65	17.47	-	-
Strawberries	2.56	1.12	-	-	11.76	5.33	-	-	-	-
Annelids	2.56	0.69	-	-	5.88	0.92	-	-	4.17	1.87
Shell fragments	3.85	0.57	10.00	1.08	-	-	5.88	1.10	-	-
Mucus/indet.	24.36	15.81	-	-	-	-	47.06	34.61	45.83	38.03

### 3.3 Parasite occurrence

**Fig. 2:**
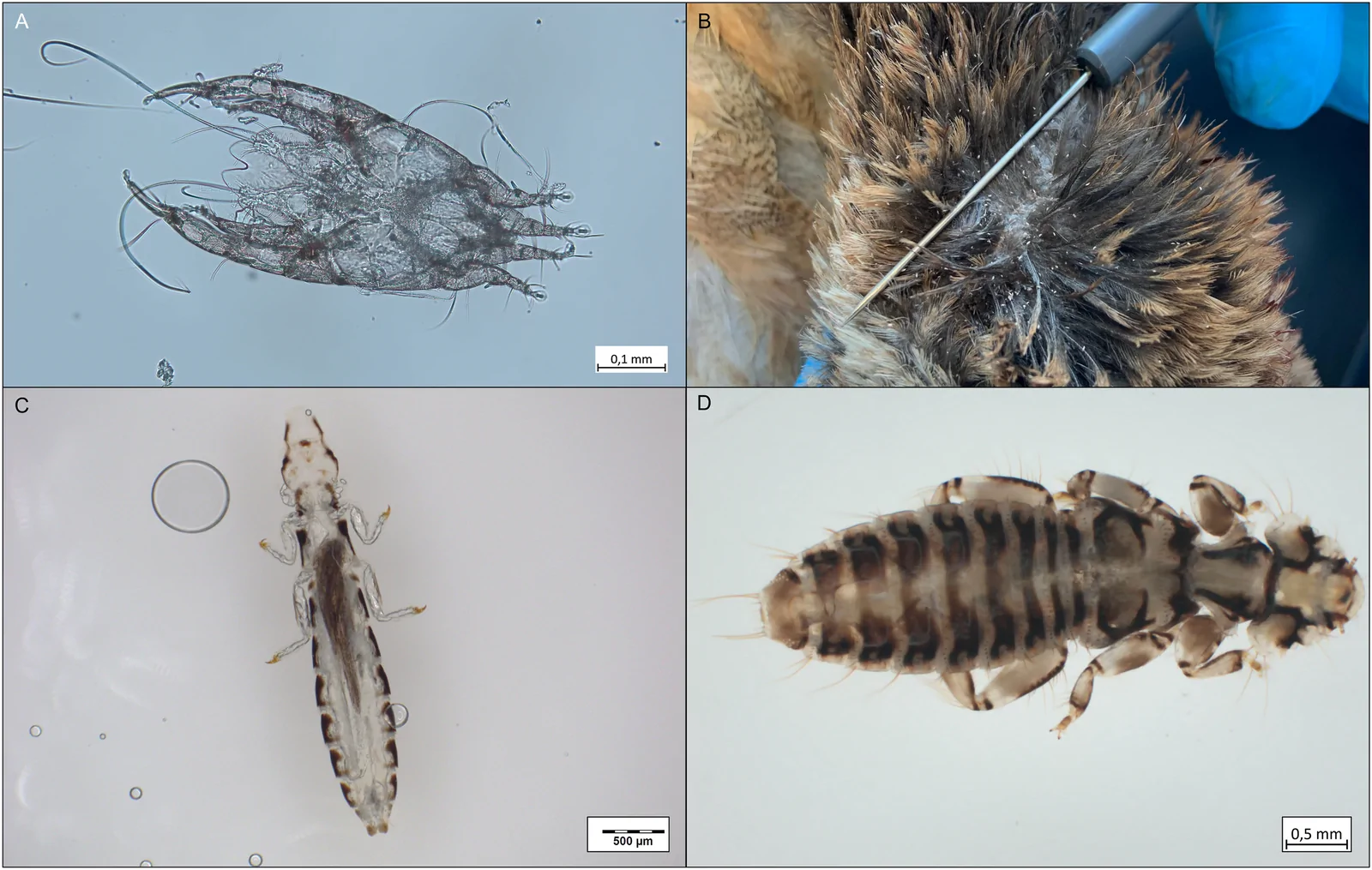
Examples of ectoparasites found in examined Egyptian geese, A: specimen of Ingrassiinae sp.; B: Severe infestation of Ingrassiinae sp. in plumage of an Egyptian goose; C: *Anaticola asymmetricus*; D: *Trinoton alopochen*.

During the external examination of the animals, a total of six species of ectoparasites were identified: one species of tick, one species of mite, and four species of lice (Table 4). *Anaticola asymmetricus* (Fig. 2C) was the most common, with an overall prevalence of P = 75.00%, followed by *Anatoecus dentatus* (P = 19.00%) and *Holomenopon tadornae* (P = 7.00%). *Trinoton alopochen* (Fig. 2D) was identified only once on an Egyptian goose during the entire study period. In addition, a mite species from the subfamily Ingrasiinae was identified (Fig. 2A and B). Among the ectoparasites, this species was found to have the highest infestation intensity in the plumage of a male Egyptian goose (maxI = 1100, mI = 222.20). The tick species *Ixodes frontalis* was identified on two of the Egyptian geese examined, with one individual found on each (P = 2.00%).

**Table 4:** Parasitological calculations for Prevalence (P [%]), mean and maximum Intensity (mI & maxI) and mean abundance (mA) of detected ecto- and endoparasites in examined Egyptian geese for the entire sampling period and each individual sampling year; * = first description for *Alopochen aegyptiaca* in Europe; ° = first description for *A. aegyptiaca* in Germany; ^p^ = human pathogenic.

		Species	Value	2021 - 2025	2021/2022	2022/2023	2023/2024	2024/2025
**Ectoparasites**	Arachnida	Ixodes frontalis*	P [%]	2.00	4.00	4.00	-	-
			mI	1.00	1.00	1.00	-	-
			maxI	1.00	1.00	1.00	-	-
			mA	0.02	0.04	0.04	-	-
		Ingrassiinae sp.	P [%]	10.00	-	24.00	4.00	12.00
			mI	222.20	-	3.17	101.00	700.67
			maxI	1100.00	-	8.00	101.00	1100.00
			mA	22.22	-	0.76	4.04	84.08
	Insecta	*Anaticola asymmetricus°*	P [%]	75.00	52.00	92.00	72.00	84.00
			mI	9.16	5.08	16.83	7.06	5.10
			maxI	55.00	42.00	55.00	33.00	35.00
			mA	6.87	2.64	15.48	5.08	4.28
		*Anatoecus dentatus°*	P [%]	19.00	12.00	36.00	12.00	16.00
			mI	6.16	1.00	5.22	0.75	16.00
			maxI	36.00	1.00	23.00	1.00	36.00
			mA	1.17	0.12	1.88	0.12	2.56
		*Holomenopon tadornae°*	P [%]	7.00	-	8.00	12.00	8.00
			mI	3.00	-	5.00	4.25	1.00
			maxI	8.00	-	8.00	8.00	1.00
			mA	0.21	-	0.40	0.68	0.08
		*Trinoton alopochen°*	P [%]	1.00	-	-	4.00	-
			mI	1.00	-	-	1.00	-
			maxI	1.00	-	-	1.00	-
			mA	0.01	-	-	0.04	-
**Endoparasites**	Digenea	*Echinostoma revolutum*^*p*^	P [%]	14.00	8.00	8.00	20.00	20.00
			mI	1.79	1.00	2.50	2.40	1.20
			maxI	7.00	1.00	3.00	7.00	2.00
			mA	0.25	0.08	0.20	0.48	0.24
		Notocotylus attenuatus*	P [%]	19.00	16.00	32.00	12.00	16.00
			mI	9.37	1.50	13.75	5.00	11.75
			maxI	53.00	3.00	53.00	7.00	17.00
			mA	1.78	0.24	4.40	0.60	1.88
	Cestoda	*Fimbriaria fasciolaris°*	P [%]	1.00	4.00	-	-	-
			mI	1.00	1.00	-	-	-
			maxI	1.00	1.00	-	-	-
			mA	0.01	0.04	-	-	-
		*Diorchis* sp.*	P [%]	4.00	8.00	-	-	8.00
			mI	1.25	1.00	-	-	1.50
			maxI	2.00	1.00	-	-	2.00
			mA	0.05	0.08	-	-	0.12
		Cloacotaenia megalops*	P [%]	6.00	12.00	-	8.00	4.00
			mI	2.00	1.67	-	1.50	4.00
			maxI	4.00	3.00	-	2.00	4.00
			mA	0.12	0.20	-	0.12	0.16
	Nematoda	Capillaria anatis*	P [%]	5.00	12.00	-	-	8.00
			mI	1.60	1.33	-	-	2.00
			maxI	3.00	2.00	-	-	3.00
			mA	0.08	0.16	-	-	0.16
		*Echinuria uncinata*	P [%]	3.00	-	-	-	12.00
			mI	4.67	-	-	-	4.67
			maxI	12.00	-	-	-	12.00
			mA	0.14	-	-	-	0.56
		*Hystrichis tricolor*	P [%]	5.00	4.00	-	4.00	12.00
			mI	2.00	1.00	-	4.00	1.67
			maxI	4.00	1.00	-	4.00	2.00
			mA	0.10	0.04	-	0.16	0.20
		Tetrameres fissispina*	P [%]	3.00	-	-	4.00	8.00
			mI	2.00	-	-	2.00	2.00
			maxI	2.00	-	-	2.00	2.00
			mA	0.06	-	-	0.08	0.16
	Acanthocephala	*Polymorphus minutus*	P [%]	48.00	48.00	68.00	24.00	52.00
			mI	19.19	10.17	12.59	3.33	43.77
			maxI	205.00	50.00	55.00	11.00	205.00
			mA	9.21	4.88	8.56	0.80	22.76
		Filicollis anatis*	P [%]	1.00	-	-	-	4.00
			mI	1.00	-	-	-	1.00
			maxI	1.00	-	-	-	1.00
			mA	0.01	-	-	-	0.04

**Fig. 3:**
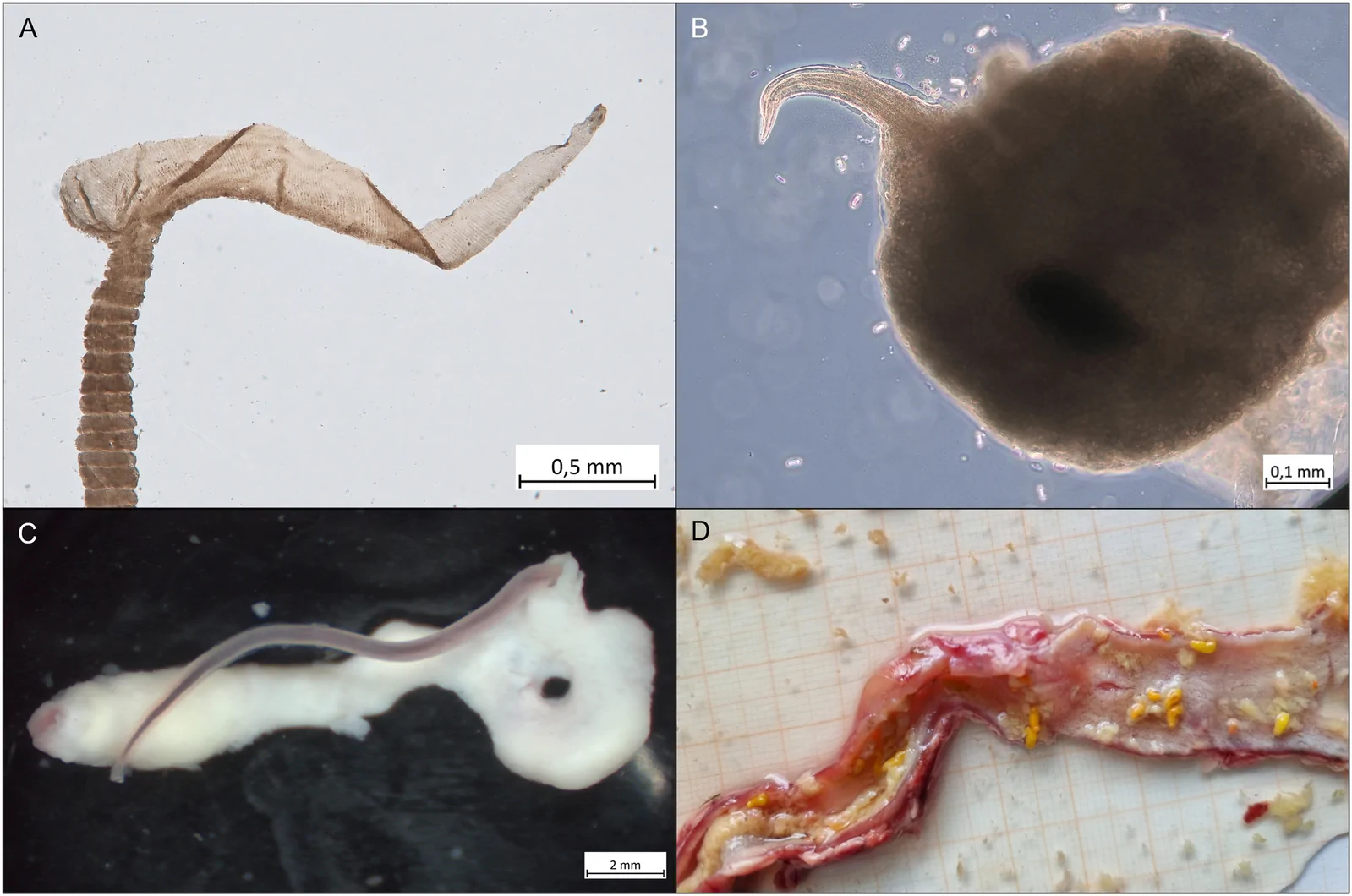
Examples of endoparasites found in examined Egyptian geese, A: pseudoscolex of *Fimbriaria fasciolaris*; B: *Tetrameres fissispina* female; C: calcified tubular structure with specimen of *Hystrichis tricolor* removed from the proventriculus; D: *Polymorphus minutus* adhering to the intestinal wall.

During the necropsy and examination of the internal organs, a total of eleven species of endoparasites (two Digenea, three Cestoda, four Nematoda, and two Acanthocephala species) were identified (Table 4). The two Digenea species, *Echinostoma revolutum* and *Notocotylus attenuatus*, occurred frequently, with P = 14.00% and P = 19.00%, respectively. Less common were the three Cestoda species, *Fimbriaria fasciolaris* (P = 1.00%) (Fig. 3A), *Diorchis* sp. (P = 4.00%) and *Cloacotaenia megalops* (P = 6.00%). The two nematode species, *Capillaria anatis* and *Hystrichis tricolor* (Fig. 3C), were found in P = 5.00% of the examined animals each, while *Echinuria uncinata* and *Tetrameres fissispina* (Fig. 3B) were found in P = 3.00% of the examined animals each. The most prevalent species was the acanthocephalan *Polymorphus minutus* (Fig. 3D), which was identified in nearly half of the Egyptian geese examined (P = 48.00%) and also reached a maximum intensity of 205 individuals in a female Egyptian goose. Finally, *Filicollis anatis* was identified in one of the Egyptian geese examined. Due to insufficient reference sequences in the NCBI database, only *E*. *revolutum*, *C*. *anatis*, *H*. *tricolor*, *T*. *fissispina*, and *P*. *minutus* could be genetically verified down to species level, for *N*. *attenuatus*, genetic evidence of only the genus could be provided (Table 1).

While eleven parasite species were identified in each of the study years 2021/2022 and 2023/2024, eight species were identified during the 2022/2023 study period. The 2024/2025 study year showed the greatest diversity, with fourteen parasite species including four ectoparasites and ten endoparasites (Table 4). Four parasite species, the feather lice *T*. *alopochen* and the endoparasites *F*. *fasciolaris*, *E*. *uncinata*, and *F. anatis*, were detected in only one study year each. The two ectoparasite species, *A. asymmetricus* and *A. dentatus*, as well as the endoparasite species *E. revolutum*, *N. attennuatus*, and *P. minutus*, were detected in all four study years. Differences in the prevalence of the various species were also observed here. The two feather lice, *A. asymmetricus* and *A. dentatus*, were most prevalent in the 2022/2023 study year, although *A. asymmetricus* showed higher infestation rates throughout the entire study period. In the same year, *N. attenuatus* and *P. minutus* also showed the highest prevalence, while *E. revolutum* was found more frequently in the last two study years.

## 4 Discussion

### 4.1 Morphometric data

The morphometric data revealed no unusual findings and were consistent with the results of previous studies (e.g. Ndlovu et al., 2010; Huysentruyt et al., 2020; Fischer et al., 2023b). Throughout the entire study period, the morphometric data for male Egyptian geese showed higher values than those for females. This confirms the previously known sexual dimorphism (Baker, 1993; Huysentruyt et al., 2020).

### 4.2 Feeding ecology

Crop contents are particularly well-suited for feeding ecology studies in birds, especially since food items are still intact and thus easy to identify (Jordan, 2005). In this study and across all study periods, corn, grains, and grasses accounted for the highest proportions. These food components serve as sources of important nutrients for Egyptian geese, particularly in winter (McLandress and Raveling, 1981; Halse, 1984). In 2021, the Wetterau district in Hesse recorded the highest silage corn yield (584.9 dt per hectare) since 2010 (HSL, 2026). During this sampling year (2021/2022), the diet of the Egyptian geese consisted mainly of corn (F = 95.00%, W = 94.50%) (Fig. 1), which could indicate a preference for particularly abundant and easily accessible resources. The excessive consumption of a single food item, such as corn, likely occurred only for a short period of time, while it was the most abundant food source. Otherwise, nutritional deficiencies might occur due to a lack of dietary variety and insufficient nutrient intake (e.g. Harrison and McDonald, 2006; Murphy, 1994). Since the animals were sampled in locations that included corn fields, or were hunted directly in those fields, the high amount of corn found in their crops was to be expected. Studies show that in certain areas, the Egyptian geese's specialization in specific food resources has resulted in crop losses (e.g. Mangnall and Crowe, 2001, 2002; Huysentruyt et al., 2020). Such effects are particularly evident when large numbers of Egyptian geese feed on this food resource over an extended period of time and should therefore be taken into account in future management of this invasive species (e.g. Kolbe, 1990; Maclean, 1997; Mangnall and Crowe, 2001, 2002; Gyimesi and Lensink, 2012; Huysentruyt et al., 2020). The detection of strawberries in one of the study years could be attributed to the use of anthropogenic resources, since the sampling date did not coincide with the ripening period of strawberries. This should be considered, particularly in the case of domestic poultry raised in free-range conditions where there is possible contact with wild animals. Furthermore, the detection of earthworms and mussel fragments indicates that Egyptian geese, which are primarily herbivorous, also consume animal-based food components, which has already been mentioned in some sources (Mangnall and Crowe, 2002; Huysentruyt et al., 2020).

### 4.3 Ecto- and endoparasite fauna

Throughout the entire sampling period, a diverse fauna of ecto- and endoparasites was detected in the Egyptian geese studied, although infestation rates were low in some cases. The tick species *I*. *frontalis* was identified as a typical avian ectoparasite; due to its toxic saliva, this species can cause symptoms of poisoning in its hosts, also known as “avian tick-related syndrome” (TRS) (e.g. Monks et al., 2006; Deplazes et al., 2021). This tick species primarily infests species belonging to the order Passeriformes, its occurrence in Egyptian geese can be explained primarily by the use of the same habitats, such as bushes or scrub (e.g. Chastel et al., 1991; Monks et al., 2006). In Germany, this tick species appears to be more widespread than previously assumed, particularly in urban areas (Drehmann et al., 2019), but has not yet been described as a parasite of the Egyptian goose.

In addition, feather mites from the family Xolalgidae (subfamily Ingrassiinea) (Fig. 2A) were identified with varying prevalences. These mites live as obligate permanent ectoparasites in the plumage of various waterbirds (Gaud and Atyeo, 1996; Dabert and Mironov, 1999). Although they do not feed on the hosts’ living tissue, severe infestation can have negative effects on the affected host due to itching and skin irritation (Gaud and Atyeo, 1996) (Fig. 2B).

Of the four identified species of feather lice, *A*. *asymmetricus* (Fig. 2C) was the most common species, with an overall prevalence of 75.00%. This species occurred at high prevalences in all study years. In the 2022/2023 study year, which had the fewest parasite species overall, it showed the highest prevalence with P = 92.00%. The various species of the genus *Anaticola* appear to be highly host-specific and differ from one another only in specific characteristics such as genital structure (Eichler and Vasjukova, 1980). This feather louse species is a typical and well-known ectoparasite of the Egyptian goose and has already been documented in studies from Belgium, Russia, and Hungary (Lapage, 1961; Eichler and Vasjukova, 1980; Hellenthal et al., 2004; Vas et al., 2012).

*Anatoecus dentatus* was also detected in all four years of the study, with the highest prevalence (P = 36.00%) also occurring in the 2022/2023 study year. Species of the genus *Anatoecus* primarily infest ducks, geese, swans, and flamingos (e.g. von Kéler, 1960; Price et al., 2003), six species are known worldwide (Grossi et al., 2014). While some species are relatively host-specific, more than 60 host species are known for *A*. *dentatus* (Grossi et al., 2014). In Germany, for example, this feather louse species has already been described for the mallard *Anas plathyrynchos* (Mey, 2003). It is also known as a parasite of the Egyptian goose from Sudan, as well as from Belgium and Hungary (e.g. von Kéler, 1960; Hellenthal et al., 2004; Vas et al., 2012).

Another feather louse species*, H*. *tadornae*, was detected in three of the four years of the study. Species of the genus *Holomenopon* parasitize birds of the order Anseriformes, primarily ducks, swans, and geese (Price, 1971). The parasitization of Egyptian geese by *H*. *tadornae* has been described in various countries on the African continent; however, this study represents the first documented case of the species in Egyptian geese in Europe (e.g. Price, 1971; Price et al., 2003).

At least, *T*. *alopochen* (Fig. 2D) was detected in only one of the animals examined. Species of the genus *Trinoton* are very easy to distinguish from other feather lice species due to their size (Eichler and Vasjukova, 1981). This species is host-specific to Egyptian geese (Eichler and Vasjukova, 1981).

To date, only limited data are available on the ectoparasite fauna of the Egyptian goose in Europe. Consequently, due to the four first records for Germany and one for Europe identified (Table 4), the present study significantly expands the known European ectoparasite spectrum of the Egyptian goose. Future studies should continue to include surveys of the ectoparasite fauna, as these organisms can often act as vectors for pathogens (Deplazes et al., 2021). This applies, for example, to the species *I. frontalis*, in which various species of *Borrelia* and *Rickettsia* have already been detected (e.g. Estrada-Peña et al., 1995; Remesar et al., 2023).

Over the entire study period, two species of trematodes were identified. *Echinostoma revolutum* is found in Asia, Europe, North and South America, and the South Pacific region, and uses aquatic lung snails as both its first and second intermediate hosts within its life cycle. In addition, mussels, frogs, and turtles are infected as second intermediate hosts while the definitive hosts of this species include various species of birds and mammals (Kanev, 1994; Chai, 2019). In Germany, this *Echinostoma* species has already been studied by Kanev (1994) and is among the better-known and more widespread species. It has also been described in Germany for the Egyptian goose by Fischer et al. (2023b), however, a lower prevalence of 7.90% was observed among the 114 animals examined. In the present study, this digenean species was detected in all four years of the study and showed an overall prevalence of P = 14.00%. Due to their lifestyle and, consequently, their consumption of intermediate hosts containing metacercariae, water-associated bird species are at a higher risk of infection than other bird species (Deplazes et al., 2021).

*Notocotylus attenuatus* was also identified in all four years of the study, with the highest prevalence (P = 32.00%) in 2022/2023. This species primarily parasitizes the cecum of birds and has a diheteroxenous life cycle (Lapage, 1961; Alexander and McLaughlin, 1997; Jirsa et al., 2021). Various species of aquatic lung snails serve as intermediate hosts (Harper, 1929; Yamaguti, 1938; Jirsa et al., 2021). This species does not exhibit high specificity for either intermediate or definitive hosts, which is why this species is widely distributed (e.g. Atkinson et al., 2008; Jirsa et al., 2021). This parasite species is primarily known from anatids such as the mallard (e.g. Prüter et al., 2018; Jirsa et al., 2021). In Germany and Luxembourg, eggs of the genus *Notocotylus* have already been identified in fecal samples from Egyptian geese, which, however, have not yet been identified up to species level (Atkinson et al., 2008; Fischer et al., 2023b).

Of the identified cestodes, *C*. *megalops* was the most prevalent species and was detected in three of the four years of the study. This species is found worldwide and is a well-known parasite that occurs in the cloaca of waterbirds, particularly those of the order Anseriformes (Woodall, 1977; Nowak et al., 2011; Guo, 2016). Ostracods serve as intermediate hosts in its life cycle. In the definitive host, the parasite does not appear to have any significant negative effects (Green et al., 2011; Guo, 2016). The genus *Cloacotaenia* had already been detected in Germany by Fischer et al. (2023b) in 2.60% (N = 114) of the Egyptian geese examined.

The cestode *F*. *fasciolaris*, which was identified during one year of the study (Fig. 3A), belongs to the family Hymenolepididae. Species of the genus *Fimbriaria* parasitize the small intestines of various bird species, particularly those of the order Anseriformes (Muniz-Pereira and Amato, 1998; Greben et al., 2023). This genus is clearly distinguished from other cestodes by specific morphological characteristics. The scolex is very small and features four suckers as well as a rostellum with a ring of ten hooks. The anterior portion of the strobila is modified into a pseudoscolex, which assists the scolex in attaching to the intestinal wall (Wolffhügel, 1900; Muniz-Pereira and Amato, 1998; Greben et al., 2023). *Fimbriaria fasciolaris* is distributed worldwide and its intermediate hosts include small crustaceans, such as species of *Cyclops* or *Diaptomus*, which are ingested by the definitive host during feeding (Deplazes et al., 2021). It has already been documented that Egyptian geese are among the range of definitive hosts (Muniz-Pereira and Amato, 1998).

Also belonging to the family Hymenolepididea is the tapeworm *Diorchis* sp, which primarily infects ducks and geese, but also other bird species (Schmäschke, 2014; Deplazes et al., 2021). In Europe the genus *Diorchis* has already been described as parasitizing mallards in Austria, Poland, and Germany (e.g. Kavetska et al., 2008; Prüter et al., 2018; Jirsa et al., 2021).

Within the phylum Nematoda, the hairworm *C*. *anatis* as well as the nematodes *E*. *uncinata*, *H*. *tricolor*, and *T*. *fissispina* were identified. *Capillaria anatis* parasitizes the lower digestive tract, primarily the cecum of birds in the orders Anseriformes and Galliformes, and thus has a broad host range (Atkinson et al., 2008; Tamaru et al., 2015). The life cycle of many *Capillaria* species is direct, which can lead to high infection rates, particularly among commercial birds kept in confined spaces (e.g. Atkinson et al., 2008; Zajac and Conboy, 2012). This hairworm has already been detected in mallards in Poland (Kavetska et al., 2008). Studies of Egyptian geese in Germany have so far detected *Capillaria* eggs in two of the feces samples examined (N = 148) (Fischer et al., 2023b).

*Echinuria uncinata* primarily parasitizes the proventriculus or the small intestinal wall of waterfowl, small crustaceans, such as various *Daphnia* species, serve as intermediate hosts (Deplazes et al., 2021). The fact that Egyptian geese in Europe are part of the host range of this nematode species has already been described by Fischer et al. (2023b). In the present study, this species was identified only in the most recent year of the study.

*Hystrichis tricolor* was present in three of the four years of the study. This species parasitizes the proventriculus of ducks, geese, and fish-eating birds and has previously been detected in Egyptian geese, even in Germany (e.g. Farias and Canaris, 1986; Kavetska et al., 2012; Gharagozlou et al., 2019; Deplazes et al., 2021; Fischer et al., 2023b, 2023c). In the parasite's life cycle, various species of earthworms serve as intermediate hosts, and fish are also highly likely to act as paratenic hosts (Hendricks et al., 1969). A severe infestation can cause extreme lesions and, through the formation of granulomas and the development of a calcified tubular structure, put pressure on other organs, such as the crop or the trachea, thereby causing further harm to the host (e.g. Fischer et al., 2023c) (Fig. 3C).

Nematodes of the genus *Tetrameres* are found worldwide and parasitize the glands of the proventriculus in a wide variety of species, primarily waterbirds. Various species of arthropods serve as intermediate hosts in the indirect life cycle of *T*. *fissispina* (Atkinson et al., 2008; Zajac and Conboy, 2012; Schmäschke, 2014; Carrera-Játiva et al., 2020; Deplazes et al., 2021). Individuals of this genus exhibit distinct sexual dimorphism. While males have a body structure typical of nematodes, females are characterized by a greatly enlarged mid-body region, which houses the large uterine tubes (Fig. 3B). The females are deeply embedded in the glands of the proventriculus, whereas the males may either be located in the glands as well or float freely in the lumen (Atkinson et al., 2008; Schmäschke, 2014). This nematode species has previously been detected in Europe in species such as wigeons, mallards, and rock doves (e.g. Carrera-Játiva et al., 2020).

The acanthocephalan species *P*. *minutus* (Fig. 3D) was the most prevalent endoparasite species, nearly half of the Egyptian geese examined were infested. Furthermore, it was detected throughout the entire study period, with the highest prevalence observed in the 2022/2023 study year. Species of the genus *Polymorphus* are among the most common acanthocephalan species in waterbirds (Atkinson et al., 2008). *Polymorphus minutus* primarily uses various amphipod species of the genus *Gammarus* as intermediate hosts, which are also widespread (Hynes and Nicholas, 1963; Atkinson et al., 2008). It had previously been detected in Egyptian geese in a German study (N = 114) with a prevalence of 7.90% (Fischer et al., 2023b). The present study shows a higher overall prevalence of 48.00%. This could be related to the presence of intermediate hosts, in which the parasite can survive for several months and thus be dispersed over long distances in water bodies (Hynes and Nicholas, 1963). While the 2023 study examined both urban and rural areas in Germany and Luxembourg, the focus of this study was on a rural region characterized by agricultural land (Wetterau), where the presence of gammarid intermediate hosts with, in some cases, high infestation rates of *P. minutus* has been confirmed (e.g. Kochmann et al., 2023). A severe infestation can have a tremendous impact on the definitive host, especially since the intestinal wall can be severely damaged by the hook-tipped proboscis (e.g. Hynes and Nicholas, 1963) (Fig. 3D).

Lastly, a specimen of the acanthocephalan species *F*. *anatis* was identified in a female Egyptian goose. This species also parasitizes the small intestine of waterfowl, primarily ducks and geese, and uses arthropods, particularly isopods, as intermediate hosts in its life cycle. Fish are suspected to serve as paratenic hosts (Zajac and Conboy, 2012; Schmäschke, 2014; Deplazes et al., 2021). Known primarily as a parasite of the mallard (Kavetska et al., 2008; Prüter et al., 2018; Jirsa et al., 2021), this finding extends the host spectrum for the parasite in Europe.

Overall, the examination of internal organs for endoparasites revealed six European and one German first records for the Egyptian goose (Table 4), thereby clearly expanding the known range of parasites associated with this invasive species.

### 4.4 Annual changes in the parasite spectrum

A diverse range of parasite species was observed across the individual study years, although individual parasite species occurred only in small numbers. Species detected in all four study years included *A*. *asymmetricus* and *A*. *dentatus*, as well as *E*. *revolutum*, *N*. *attenuatus*, and *P*. *minutus*. All of the species mentioned are known as parasites of waterbirds in both the original and the newly colonized ranges of the Egyptian goose, with *A. asymmetricus* and *A. dentatus* considered typical parasites of the Egyptian goose (e.g. von Kéler, 1960; Eichler and Vasjukova, 1980; Hellenthal et al., 2004; Vas et al., 2012; Fischer et al., 2023b). The fact that these species were present at comparatively high prevalences throughout the entire study period distinguishes them as potential key species for the Egyptian goose in the study area. Except for *E. revolutum*, all key species exhibited their highest annual prevalence rates during the 2022/2023 study period. At the same time, this study year was characterized by the lowest parasite diversity, which could indicate a dominance of these key species (e.g. Nentwig et al., 2009; Krauβ and Bärlocher, 2025). Overall, the parasite spectrum of the Egyptian geese studied shows significant overlap with that of the native mallard, which could be explained by their use of similar water-rich habitats and, in part, comparable lifestyles (e.g. Mey, 2003; Kavetska et al., 2008, 2012; Prüter et al., 2018; Jirsa et al., 2021).

The detection of the endoparasitic key species *E. revolutum*, *N. attenuatus*, and *P. minutus*, whose life cycles include obligate intermediate hosts, together with the results of the dietary analysis, suggests that the predominantly herbivorous Egyptian geese also consume animal-based food components (Huysentruyt et al., 2020; Mangnall and Crowe, 2002). These findings also underscore the Egyptian goose's association with water bodies and the importance of aquatic organisms as a food resource. The two digenean species use freshwater snails as their first intermediate hosts, while *E. revolutum* can also use mussels, among others, as second intermediate hosts (Harper, 1929; Yamaguti, 1938; Kanev, 1994; Chai, 2019). Although no corresponding animal food components were detected, with the exception of mussel fragments in the study years 2021/2022 and 2023/2024, the detection of the parasite species provides indirect evidence of the ingestion of their intermediate hosts (e.g. Williams et al., 1992; Marcogliese and Cone, 1997; Marcogliese, 2004, 2005; Peter et al., 2024). The relevant intermediate hosts could inadvertently enter the digestive tract along with gastroliths ingested from the water or, in case of mussel fragments, even were consumed as grit to aid digestion or as a source of calcium (e.g. Reynolds and Perrins, 2010; Schwoerbel and Brendelberger, 2022; Thomas et al., 1977; Wink, 2025). The high prevalence of *P. minutus* also suggests that the ingestion of aquatic invertebrates such as gammarids, which act as intermediate hosts, plays a significant role in the feeding ecology of the Egyptian goose alongside plant matter and anthropogenic food resources such as corn (Hynes and Nicholas, 1963; Atkinson et al., 2008). Infected intermediate hosts are presumably ingested unintentionally along with aquatic plants in which they reside (e.g. Eggers and Martens, 2001; Schwoerbel and Brendelberger, 2022). The high prevalence rates could be facilitated in particular by the fact that *P. minutus* does not follow a distinct seasonal occurrence pattern, can persist in the intermediate host for several months, and can survive as an adult parasite in the definitive host for more than 165 days (Hynes and Nicholas, 1963; Romanovski, 1964). This allows for repeated infections throughout the year, which can lead to high prevalence rates over the long term.

Other identified species of endoparasites also use animal intermediate hosts. The two cestode species *C*. *megalops* and *F*. *fasciolaris*, the nematodes *E*. *uncinata* and *T*. *fissispina* and the acanthocephalan *F*. *anatis* use various arthropods as intermediate hosts in their life cycles (Green et al., 2011; Guo, 2016; Deplazes et al., 2021). The nematode *H*. *tricolor* uses earthworms as intermediate hosts in its life cycle (Hendricks et al., 1969). Its detection in three of the four sampling years can therefore be attributed to the ingestion of infected earthworms and is consistent with the detection of annelids in the food samples from the study years 2022/2023 and 2024/2025. The occurrence of these intermediate hosts is likely subject to seasonal fluctuations, as has been described for earthworms, for example. Earthworms exhibit fluctuating activity, due to changing soil temperatures and soil moisture (Potvin and Lilleskov, 2017) and are presumably ingested less frequently by potential definitive hosts such as the Egyptian goose. While a dietary analysis alone can only provide a snapshot of a specific time, the inclusion of parasite fauna can also yield insights into the general dietary patterns and past feeding events of the studied animals (e.g. Marcogliese, 2005; Baker et al., 2024). Various factors, such as the state of the ecosystem, the presence of potential intermediate and definitive hosts, and prevailing climatic conditions, influence the available food supply and thus also the extent of parasitism (Schnieder, 2006). Consequently, the composition of the parasite fauna as well as the diet of the studied animals may differ, even if sampling took place at the same time of year. Nevertheless, the present study provides a comprehensive overview of the feeding ecology and parasite infestation of the Egyptian goose in the study area and expands knowledge about its parasite fauna in Europe.

## CRediT authorship contribution statement

**Anna V. Schantz:** Writing – review & editing, Writing – original draft, Visualization, Validation, Methodology, Investigation, Formal analysis, Data curation, Conceptualization. **Robin Stutz:** Writing – review & editing, Writing – original draft, Visualization, Validation, Methodology, Investigation, Formal analysis. **Anne Steinhoff:** Writing – review & editing, Writing – original draft, Validation, Investigation. **Norbert Peter:** Writing – review & editing, Writing – original draft, Supervision, Methodology, Investigation, Funding acquisition, Conceptualization. **Anna Thomas:** Writing – review & editing, Writing – original draft, Visualization, Investigation. **Dorian D. Dörge:** Writing – review & editing, Writing – original draft, Visualization, Funding acquisition. **Sven Klimpel:** Writing – review & editing, Writing – original draft, Validation, Supervision, Resources, Project administration, Methodology, Funding acquisition, Data curation, Conceptualization.

## Data availability

The raw data on which the calculations are based and which support the conclusions of this article will be made available by the authors without reservation on request.

## Competing interests

The authors declare no competing interests.
